# Systemic inflammatory response to inhaled endotoxin does not correlate with airway response

**DOI:** 10.1186/s12931-019-1227-3

**Published:** 2019-11-12

**Authors:** Amika K. Sood, Allison J. Burbank, Michael Lawson, Haibo Zhou, Heather B. Wells, David B. Peden, Michelle L. Hernandez

**Affiliations:** 1 Center for Environmental Medicine, Asthma, and Lung Biology, University of North Carolina, 104 Mason Farm Rd #CB7310, Chapel Hill, NC 27599-7310 USA; 2 Division of Allergy, Immunology and Rheumatology, Department of Pediatrics, University of North Carolina, 030 MacNider Hall CB#7231, 333 S Columbia St, Chapel Hill, NC 27599 USA; 3 Department of Biostatistics, Gillings School of Global Public Health, University of North Carolina, 3101 McGavran-Greenberg Hall CB#7420, 135 Dauer Drive, Chapel Hill, NC 27599 USA

**Keywords:** Endotoxin, Neutrophils, Airway inflammation, Systemic inflammation

## Abstract

**Background:**

Endotoxin is a component of particulate matter linked to respiratory disease. Our group has shown that experimental endotoxin inhalation challenge reproducibly triggers neutrophilic inflammation in the airways and in peripheral blood. Sputum induction is currently the only available method for assessing airway neutrophilia but is laborious and time-consuming. This analysis examined the correlation between systemic and airway inflammatory responses to endotoxin to determine if peripheral blood could serve as a surrogate marker for neutrophilic airway inflammation.

**Methods:**

We conducted a retrospective study of 124 inhaled endotoxin challenges conducted at our center using 20,000 endotoxin units (EU) of Clinical Center Reference Endotoxin (CCRE). Venipuncture and induced sputum samples were obtained at baseline and 6 hours after completion of endotoxin challenge. The relationship between change in sputum neutrophils (post-challenge – baseline) and change in peripheral blood neutrophils (post-challenge – baseline) was assessed using Spearman’s correlation analyses.

**Results:**

Inhaled endotoxin induced a significant increase in mean sputum percent neutrophils and peripheral blood absolute neutrophil counts in healthy adults with or without mild asthma, but no significant correlation was found between airway and systemic neutrophilia (*r* = 0.13, *p* = 0.18). Stratification by degree of airway neutrophil response and by atopic or asthmatic status did not change the results.

**Conclusions:**

Inhalation challenge with endotoxin safely and effectively induces airway neutrophilic inflammation in most individuals. Increases in endotoxin-induced peripheral blood neutrophils do not correlate well with airway responses and should not be used as a surrogate marker of airway inflammation.

## Background

Endotoxin, a component of gram negative bacteria, is found ubiquitously in the environment in both domestic and occupational settings as well as in ambient air particulate matter [1]. Exposure to endotoxin has been linked to respiratory disease, including occupational lung disease [1] and asthma [2]. Endotoxin is a strong activator of the innate immune response through its binding to Toll-like receptor 4 on the surface of airway macrophages, inducing a potent pro-inflammatory cytokine response with subsequent recruitment of granulocytes [1]. As both we [3, 4] and others [5] have demonstrated, endotoxin inhalation has both airway and systemic inflammatory effects, with increased neutrophils in induced sputum as well as increased peripheral blood neutrophils. Whether systemic neutrophilia results from airway “spillover” of neutrophils into peripheral blood versus neutrophil recruitment to the blood by systemically release inflammatory cytokines remains unclear.

Our group has long utilized inhaled endotoxin challenge as a model of neutrophilic airway disease and for studies of novel anti-inflammatory treatments. Treatment efficacy is best assessed through the examination of the airway inflammatory cellular milieu via induced sputum sampling before and after inhaled endotoxin challenge. However, in our experience over the last three decades, healthy volunteers are only able to provide adequate sputum samples about two thirds of the time and those with underlying respiratory disease even less so. This highlights the need for a more readily attainable biomarker of endotoxin-induced airway inflammation. We examined the correlation between systemic and airway inflammatory responses to endotoxin to determine if peripheral blood neutrophilia could serve as a surrogate marker for airway inflammation.

## Methods

We conducted a retrospective study of 124 inhaled endotoxin challenges conducted at our center using 20,000 endotoxin units (EU) of Clinical Center Reference Endotoxin (CCRE) obtained from the National Institutes of Health. All doses were prepared by the Investigational Drug Service and inhaled by subjects as a nebulized preparation using a DeVilbiss Ultraneb 99 ultrasonic nebulizer (DeVilbiss, Port Washington, NY). Venipuncture and induced sputum samples were obtained at baseline and 6 hours after completion of the challenge. Wilcoxon signed rank test was used for post-challenge versus pre-challenge comparisons of sputum percent neutrophils (%PMNs) and of blood absolute neutrophil count (ANC) and blood %PMNs. Spearman’s correlation analysis was then performed to assess the relationship between the change in sputum PMNs (post-challenge – pre-challenge) and the change in peripheral blood PMNs (post-challenge – pre-challenge). Because of the substantial inter-subject variability in sputum absolute cellular counts, we focused our analysis on %PMNs in sputum as the primary measure of endotoxin-induced airway inflammation.

## Results

**Fig. 1:**
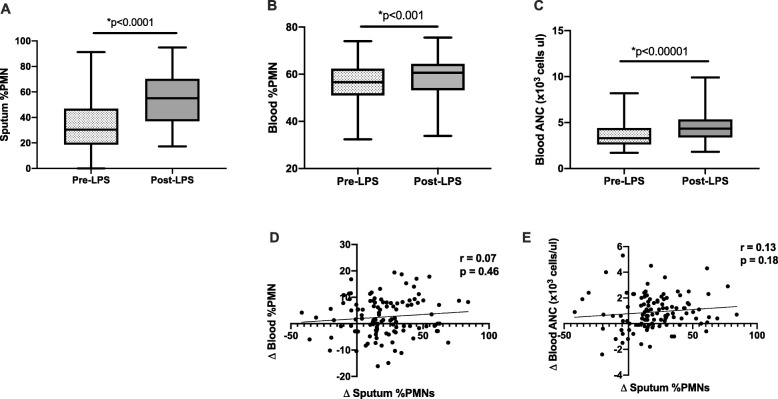
Comparison of airway (**a**) and systemic (**b**, **c**) neutrophilic responses before and after inhaled endotoxin challenge showed significant increase in neutrophilic inflammation (*N* = 124). Correlation analyses of sputum % PMNs versus blood %PMNs (**d**) and blood ANC (**e**) revealed no significant correlation

Demographic data for our sample population is presented in Table 1. Notably, our study population included both healthy volunteers as well as mild intermittent asthmatics but excluded smokers, who are exposed to higher levels of environmental endotoxin via cigarette smoke [1]. Endotoxin challenge was well tolerated without changes in lung function or measurable systemic symptoms, such as fever. Inhaled endotoxin induced a significant increase in mean sputum %PMNs from 34% ± 21 (SD) to 54% ± 20 (*p* = < 0.0001, Fig. 1a) in the total population. A modest increase was seen in mean blood %PMN (57% PMNs ±8 pre-LPS to 59% ± 8 post-LPS; *p* < 0.001; Fig. 1b) and in blood ANC (3600 cells/μl ± 1300 pre-LPS to 4500 ± 1600 post-LPS; *p* = < 0.001; Fig. 1c). However, correlation analyses failed to reveal any significant relationship between systemic and airway neutrophilic inflammation, with systemic defined by either blood %PMNs (*r* = 0.07, *p* = 0.46, Fig. 1d) or blood ANC (*r* = 0.13, *p* = 0.18, Fig. 1e).

**Table 1:** Demographic Characteristics (*n* = 124)

Age (years), median (range)	25 (19–49)
Sex (Female/Male)	81 female/43 male
Race	87 Caucasian/white
	31 African American
	4 Asian
	2 Hispanic
BMI (kg/m^2^), median (range)	24.5 (19–42)
Atopic, N (%)	33 (27%)
Asthmatic, N (%)	31 (25%)

The data were then stratified by the degree of endotoxin-induced sputum neutrophilia observed (< 10% increase vs > 10% increase in sputum % PMNs from baseline post-endotoxin challenge). This criterion has been established by our group to indicate whether a participant is sufficiently responsive to inhaled endotoxin for inclusion in proof-of-concept interventional studies [6]. Of those who experienced a > 10% increase in sputum %PMNs following endotoxin challenge (*n* = 85), no significant correlation was seen between sputum %PMNs and systemic neutrophilia defined by blood ANC (*r* = 0.11, *p* = 0.32) or by blood %PMNs (*r* = 0.07, *p* = 0.52). Stratification of the total population by atopic and asthmatic status did not uncover any significant correlation between endotoxin-induced systemic and airway neutrophilic responses (Table 2).

**Table 2:** Spearman correlation analyses stratified by asthma and atopic status

	Asthma		Atopy	
	Present (*N* = 31)	Absent (*N* = 83)	Present (*N* = 33)	Absent (*N* = 81)
Blood ANC vs Sputum %PMN				
r	−0.02	0.1	−0.13	− 0.06
p	0.93	0.34	0.48	0.58
Blood %PMN vs Sputum %PMN				
r	−0.08	0.19	0.02	0.1
p	0.71	0.28	0.93	0.37

## Discussion

We have shown that endotoxin-induced systemic inflammation does not correlate well with airway inflammation in healthy and mild asthmatic volunteers, suggesting that peripheral blood neutrophilia is not an adequate surrogate marker of endotoxin-induced airway inflammation. Other groups have similarly reported reduced accuracy of peripheral blood eosinophils as a surrogate marker of eosinophilic airway inflammation in asthma [7]. With the paucity of readily obtainable non-invasive biomarkers for neutrophilic airway disease, induced sputum remains the best sampling method to accurately detect neutrophilic inflammation in the lung. Similar to use of exhaled nitric oxide for detection of eosinophilic airway inflammation, the search is underway for sensitive biomarkers of neutrophilic airway inflammation in exhaled breath. Esther et al. found that purine concentration in exhaled breath condensate was significantly correlated with lung function in children with cystic fibrosis (a neutrophil-predominant pulmonary disease) [8].

Our study focused on the quantity of neutrophils present before and after endotoxin challenge. A limitation of our approach is that it did not provide information on pollutant-induced neutrophil activation. Future work will include an assessment of markers of neutrophil activation before and after pollutant exposure, such as myeloperoxidase, neutrophil elastase, and neutrophil extracellular traps.

In this study, we have shown that otherwise healthy adults who experience airway inflammation in response to endotoxin may or may not exhibit a commensurate increase in systemic inflammation. These findings suggest that the increase in circulating neutrophils induced by endotoxin is not simply a “spillover” of neutrophils from the airways but may result from other mechanisms such as systemic release of inflammatory cytokines that recruit neutrophils to the peripheral blood. The fact that not all subjects experienced a commensurate increase in peripheral blood neutrophilia suggest that particular biological factors may impact susceptibility to endotoxin-induced systemic inflammation, and this is a focus of ongoing work at our center. Though not the primary objective of this study, this observation provides insight into the mechanism through which endotoxin inhalation leads to systemic inflammation.

## Conclusions

Our findings support that peripheral blood sampling should not replace sputum induction for assessment of neutrophilic airway inflammation in endotoxin inhalation challenge. Reliable and accessible biomarkers are needed to decrease the reliance on laborious and invasive sampling methods for assessing neutrophilic airway inflammation.

## Data Availability

The datasets used and analysed during the current study are available from the corresponding author on reasonable request.

## Abbreviations

ANC: Absolute Neutrophil Count
CCRE: Clinical Center Reference Endotoxin
EU: Endotoxin units
PMN: Neutrophil
